# Indents

**Published:** 1909-02-15

**Authors:** 


### INDENTS.

☞ **Brief Articles of Technical or Scientific Value will receive notice and comment, Contributions invited.**

*Rules for Eating.* 1. Do not eat until a plain piece of bread or a dry cracker tastes good. 2. Chew all solid food until it is liquid and almost or quite swallows itself. 3. Sip all liquids that have taste, including soups, sodas, lemonades, etc., until all the taste is out of them. It is well worth while. Pure water for quenching thirst has no taste and may be swallowed immediately. 4. Never eat while you are worried or angry only when you are calm. Waiting for a calm mood will bring good appetite juice; without it there is poor digestion. 5. Learn and practice these four rules; eat whatever appetite calls for most loudly, and both your teeth and general health will be fine.—Horace Fletcher, in A. B. Z. of *Our Own Nutrition.*

*A case of Infection:* My attention has recently been called to a peculiar case of infection: A dentist in performing an operation for a young lady let an instrument slip and it cut her lip slightly. He thought nothing of it at the time, nor did she. In due time she had a chancre of the lip at the position of the little cut. Naturally the dentist was very much exercised and started an investigation. He was able within a few days to establish the fact that the young lady kissed a syphilitic young man on the night of the day on which her lip was cut. If the dentist had not punctured the lady's lip, probably she would not have had infection. On the other hand, if she had not kissed the young man she would certainly not have had it, as the dentist's instruments had been sterilized. If the dentist had taken the precaution to have cauterized the cut with carbolic acid and swabbed it over with alcohol, possibly she would not have been infected.—Arthur D. Black, in *Review.*

*Effects of Contraction in Inlay Casting.* Contraction of the gold will do but little harm when the inlay is intended for a simple occlusal cavity with tapering walls and rounded margins. If, however, the restoration is a mesial and distal filling, united into one piece through the occlusal surface, the contraction will prevent its going to place, and to force it means simply to spread it out at the gingival margins, like spreading a horseshoe. It cannot be corrected by grinding. In bridge work, because of the length of the piece the contraction makes it too short to reach the abutments. The normal contraction of the metal can be partially, but not wholly, overcome by pressure.—Weston A. Price, *Items of Interest,* Dec. 1908, page 932.

*Shield the Innocent:* I prefer arsenic to cocain for the removal of the dental pulp, because many patients approach the dental chair with hesitation and trepidation. Suffering endured while in this mental condition is much intensified. Nearly all stories of dental agonies reach their climax in "the way I suffered in Dr. So-and-So's office," while ten-fold more suffering endured before the doctor was consulted is seldom mentioned to their timid friends. While we cannot in every case remove the pulp painless with either drug, I find arsenic on the whole, milder, besides a portion of the pain is not endured while in the dental office. This from the standpoint of both patient and dentist is worth considering. Covering the exposed pulp after applying the treatment with a sheet of tin under the stopping, as recommended by Dr. W. T. Reeves, of Chicago, prevents pressure on the pulp and leaves a receptacle for blood, gas or serum and almost entirely eliminates pain.—Wm. Dick, Trimountain, Mich., in *Dental Review.*

*Cap and Dowel to Accurately Fit.* After loosely fitting the dowel to the enlarged pulp canal, solder to it a piece of gold plate somewhat smaller than the root end, at such a point that when the dowel is placed in the prepared canal, the plate of gold will slightly stand off from the end of the root. Then roughen the pin, run casting wax over both dowel and gold plate, and press the whole into position. The piece of plate holds the wax in shape, and prevents any displacement in removing it from the root. After carving the wax as the cap is desired to be, invest and cast. The result will be a closely fitting dowel and cap. This method will be found of especial advantage in cases where two dowels are needed.—Dr. Celia Rich, Nashville, Tenn., *Dental Cosmos*, Dec. 1908, page 1416.

*Plaster Impressions: A Separating Coating.* Dr. Eli J. Sweet, Hornell, N. Y., recommends a solution of anilin in alcohol for coating plaster impressions before making the cast, to prevent the new plaster uniting with the old. He coats the impression with the pigment, then places it in a basin of water 10 to 15 minutes, and without using varnish or oil pours the cast. It separates quite easily, and if necessary to cut the impression to facilitate its removal, the red pigment marks plainly the dividing line. As there is nothing on the surface of the impression, the cast is quite sharp. He also finds that when the cast is used for vulcanizing the rubber parts from the plaster quite clean without the use of silex.—Clinic 7th and 8th District Dental Society of New York.

*Zinc Oxid Lining For Attachment of Porcelain Inlays.* Zinc oxid heated to a temperature of about 2,800 degrees becomes hard, vitrified and granular. To two parts, by weight, of the granular zinc oxid, which is the principal constituent of cement powder, is added one part of foundation body. Give the inlay matrix a thin coating of this mixture, having it quite moist. Do not jar to place, for the coarse zinc oxid would then come to the surface and the foundation body would settle to the matrix. Place in furnace, but do not bring to the temperature required to fuse the foundation body. The coating will show a distinct yellow color when taken from the furnace, but will change to light gray as soon as cooled. Proceed with the foundation body as for ordinary inlays. Upon removing the matrix, if you find some part of the base slightly glazed, scratch it with a sharp-pointed instrument and the granules of the zinc oxid will appear. You will now have a finished inlay, having not only a roughened surface to which the cement will cling, but also one with which the liquid of the cement mix will unite chemically, and there will be actual cohesion of the inlay surface and the cement. A. M. Harrison, *Dental Review.*

*An Accurate and Safe Way of Applying Silver Salts.* Secure three sizes of smooth, flexible, canal-dressing instruments of platinum, German silver, or the so-called platinoid material. Secure a small, wide-mouthed bottle and place in the same the granular salts. Heat your instrument slightly and plunge it into the bottle, taking up a crystal of any size that you may wish to use. Your crystal, which is now only slightly attached, is passed back and forth in a flame until it is thoroughly fused on the very tip of your instrument. Having thus been fused to the end of the instrument, the salt can in full strength be safely and accurately applied to either hard or soft tissues.

Silver nitrate is preferred by the clinician if discoloration be not objectionable.

In applying salts by this method it must be kept in mind that the salts are used full strength; the tissues must therefore be protected by the use of napkins and the saliva pump. If the spot to which the salt is applied is not moist enough to gradually dissolve the fused point, it is moistened with a little damp cotton. By having flexible instruments of three different sizes, the point of fused salt can be applied in varying sizes, which allows greater accuracy. The flexible instruments can be bent to any angle desired.—A. J. Flanagan, *Dental Cosmos.*

*Orthoform—Its Effects, Properties And Uses.* It is a white crystalline powder, of weak acid reaction, slightly and very slowly soluble in water, freely soluble in alcohol, ether and chloroform, and is without odor or taste. Upon the tongue, orthoform produces at first no apparent effect, owing to its low solution; after a minute a gradual numbness is noticed, and the limited locality loses its wonted keen sensibility to pain. When exhibited in dry powder it is practically non-poisonous—internal dose 0.06 to 0.65 gm. (gr. 1-x), and may be used locally *ad libitum*, but when applied freely in solution of ointment it might induce some trifling unpleasant sequelæ. It possesses some antiseptic and anesthetic properties, and is unquestionably a most distinguished analgesic; it is accordingly indicated in severe burns and scalds, ulcers of the tongue and lips and of other parts; to relieve the pain after extraction of teeth; in pyorrhea pockets, before and after operation, and it is especially effective in reducing the interminable agony of "dry pockets." In the latter case, wash out with a syringe and warm water, then gently place in the socket a pledget of loose cotton wool holding in its meshes as much of the powder as they can carry; and the erstwhile suffering patient will be indeed truly grateful. The application need not usually be repeated for a day or two, as the beneficent effect of the drug is prolonged, mainly owing to its slow solubility.—J. S. Cassidy, *Dentist's Magazine.*

*Lower First Molar Bridge.* All sorts of methods have been devised for constructing and placing a fixed bridge where the abutment teeth incline considerably towards each other, but none, so far as the writer is aware, that did not involve considerable sacrifice of tooth tissue or imperfect adaptation or both. Dr. Hulick, of Cincinnati, seems to have solved the problem in an exceedingly simple manner. In a case where a second molar and a first bicuspid serve as pier teeth the molar is fitted with a gold shell crown in the usual manner. The bicuspid, after having its occlusal surface ground down is covered with a gold cap having a flat top to fit the ground surface. To this cap is soldered a gold tube extending into the root canal, after which it is cemented into place. A Gold cusp-end is next made of suitable length and form to occlude correctly with its antagonist and to its underside is soldered a gold wire rather short and somewhat smaller in diameter than the tube which is to receive it. The bridge is now completed and will include the molar crown, the intervening dummies and the cusp and pin of the bicuspid. In cementing it into position the molar end is tipped and placed over the molar after which the bicuspid end is brought down into place, the cement fixing the short pin or dowel in the canal tube and closing the joint between the cusp and cap. The idea is extremely simple and the result as perfect as though the abutments were parallel —Dr. Guilford, in *Garretsonian.*

*Blackening of the Tongue by Hydrogen Dioxid.* Attention has been directed by M. L. Bizard (*Les Nouvenaux remedes; The Lancet,* October 31, 1908) to the phenomenon of black tongues caused by the use of Hydrogen Dioxid as a mouth wash. He cites the case of a smoker who used a mouth wash daily for eight days consisting of a tablespoonful of hydrogen dioxid in half a glass full of warm water. A marked blackening was then found on the dorsal surface of the tongue, but on ceasing to use the mouth wash the coloration disappeared in a few days. The patient was in good health, except that an examination of the tongue showed that the lingual papillæ were hypertrophied. The same effect was produced in the case of two patients undergoing mercurial treatment by the injection of grey oil, who were in the habit of rinsing the mouth with hydrogen dioxid. It was at first thought that the color was due to the action of the hydrogen dioxid on the mercurial saliva, but hydrogen dioxid alone, as has been shown, may produce the same effect. It is remarked by the editor of The *Lancet* that, as hydrogen dioxid has now come into general use as a mouth wash, these observations are of great interest and value. Apart from the blackening of the tongue, which may result from too free a use of this preparation, the question arises whether the general employment of antiseptic mouth washes and dentifrices is advisable in healthy persons. Where decay is known to exist there is no doubt that a moderate use of antiseptic agents tends to prevent the spread of the invading organisms. But when the mouth is in a healthy condition it would seem preferable to rely on cleanliness rather than on antiseptics. The bacterial flora of the mouth may contain organisms that naturally tend to preserve it in a healthy condition, but the continued use of antiseptics is calculated to destroy favorable as well as harmful bacteria.—*York Medical Journal.*

*Mounting the detached-Post Crown on Cast Gold Base.* Prepare the root end by the ordinary method of setting such a crown,and adapt the crown to it by grinding. A perfect adaptation is not necessary, however, at this stage, although the crown and stump should approximate closely on the labial side. Grind out a small semi-circular space in the edge of the palatal side of the crown to allow a little more space between this part of the crown and the root end. The importance of this little space will be obvious as we proceed. A dowel of round platinous gold or iridio-platinum wire of suitable size is cut to proper length and is further prepared by melting fast to it near its center, a small disk of wax such as is used for inlay patterns. This wax should be sufficient in bulk to complete the adaption between the crown and the root. Oil the cut end of the crown, slightly warm the wax, and assemble the crown, wax, and dowel on the stump. Press home hard enough to make the crown and stump come into contact, or until only a film of wax remains at the closest places. Remove from the stump, chill in water, and with the wax, crown, and dowel assembled remove the surplus margin of wax with a sharp knife. The sprue wire is now inserted into the edge of the wax disk where it has enlarged into the little semi-circular space at the palatal side and the whole is again assembled on the stump to correct any defects in the adaption caused by trimming and placing the sprue wire. The wax disk and dowel are now ready to be flasked in the ordinary manner for casting, and a disk of pure gold is cast directly on the dowel. The result is a gold fixture that will take the place of the objectionably large cement line, avoid all the misfits ordinarily present, and make an adaption that will reduce the cement line to the same infinitesimal minimum that is present under a well-fitted gold or porcelain inlay.—J. G. Lane, in *Cosmos.*

*Chloroform Anesthesia and Exclusion of Part Of The Circulation.*

Since the interference with respiration and failure of the heart are so liable to occur during the administration of chloroform, any new suggestion which may diminish the frequency of these complications or aid in their treatment is deserving a trial.

About a year ago Klapp suggested tying of the extremities temporarily during the chloroform anesthesia, as in this way part of the blood was excluded from a general circulation. Thus less chloroform would be required to obtain the same amount of anesthesia, it being a well-known fact that the smaller the amount of circulating blood the less chloroform will be needed. Recently Zeigner (*Medizinishe Klinik* No. 17, 1908) proved the usefulness of Klapp's suggestion in a series of animal experiments. He actually found that much less chloroform was required to bring about the same degree of anesthesia when the animal's extremities were tied than when this was not done. He furthermore found that when animals had their extremities tied, those which had the ligatures cut immediately after the anesthetic was stopped recovered more quickly than those in which the ligatures were left in place. The less chloroform used the less likely complications are to set in during the course of an anesthesia, and as the severity of the after-effects is directly due to the amount of anesthetic which the organism has to eliminate, it is necessary to limit the quantity given as much as the exigencies of the operation and the patient will permit. In those patients in whom we fear complication or severe after-effects Klapp's suggestion would be advantageous in that less chloroform is necessary. Ziegner's experiments also demonstrate that in cutting out a part of the circulation during an anesthesia we have a valuable remedy in combating any interference with respiration and heart failure, for upon removing the ligatures blood not loaded with chloroform immediately reaches the vital centers and acts as a most powerful stimulant.—*Medical Record.*

*Banding a root so that the Gold will Restore a Perfect Contour Between Root and Crown.*

Prepare the root by beveling considerably toward the crown end. Cut a band as small as will go around the smallest part of the prepared end of the root, so that, as it is pressed to place, it will stretch to fit closely the beveled portion.

Now grind the root, with the band in position, as short as desired, then grind a porcelain crown—I prefer detached-post crowns—to fit the case, as far as length and occlusion go. Now burnish or swage a piece of No. 35 pure gold to accurately fit the end of the porcelain crown next to the band.

Then place the crown, with this gold on it, on the root with the band on it, and with the tooth in its proper position, mark where the gold band and the gold burnished over the end of the crown come together, so that both the band and the gold on the crown can be removed and caught together with a very minute piece of solder on the lingual side only, just enough to hold the two pieces together. They are then replaced on the root and the crown is put into position, and as the band and crown plate have only been slightly caught together on the lingual side, there is some latitude left in the adjustment of the band and the crown plate, which are now made perfect as to their relation to each other, although they need not necessarily fit closely together. Both band and crown plate are now slipped off and the inside of the band and the side of the crown plate, where the crown is to set, are filled with powdered asbestos mixed with water, and a little wire is twisted around to hold the parts in position. Then on the outside of the band and between the band and the crown plate flow some 22-k. gold solder, then cement the crown and the band and the post together in position on the root, but do not cement to the root yet, as when the cement is hard, the crown, band and post are removed and the band is finished down even with the crown. It is then ready to be permanently cemented upon the root.—A. J. Sawyer, in *Cosmos*.

*Packing Vulcanite in the Flask.* The old method, and that usually employed now, is defective from the fact that the flasks are not always closed exactly as they were when the investment was made. This results from the rubber not being soft enough to flow easily, as the flasks can not be handled if thoroughly heated, and the force necessary to close the flasks, especially in the old bolt flask, is so great as to force the teeth out of their original position and often cause dark joints where block teeth are used. To obviate this trouble it is only necessary to apply just enough pressure to assure us that we have enough rubber packed for the case, and then depend upon a slight but constant pressure after being placed in the vulcanizer. When the piece is run up to the heat necessary to vulcanize it the rubber becomes semi-fluid, and requires the slightest pressure to send it home. I secure these results by making a flask that from its construction must close true, and a clamp or press of spring brass, with its top depressed, and the sides bulging, or barrel shape; then by turning the screw of press onto the flasks containing the teeth until you feel a slight pressure, give it one full turn, by which operation you lift the depressed top of clamp and draw in its bulging sides, which have a tendency to return to their normal shape as rapidly as the rubber becomes soft, which it does when run up to the required heat in the vulcanizer. Have no fears of desired results, even if the flasks are not closed when put in the vulcanizer.

My own method of investing is exactly the reverse of the methods generally employed. Instead of having the teeth draw in upper part of the flask when separated, I invest in such a way as to leave both teeth and cast in the lower part with as strong an embankment of plaster around them as possible. This method necessitates a deep and large flask, with greater anterior width than most flasks possess, and insures a proper retention of the teeth in their relative relation to the cast, no matter how the flasks are closed, or even if they are not closed this method I have practiced exclusively for many years. Should it happen in running the wax from the case, that some of the teeth, or even all of them, should drop out of place it is a simple matter to replace them in their matrix formed in the plaster, and proceed with the packing. This method of investment has the merit among others of retaining the pink facing rubber just where placed, and prevents the black or other colored rubber from forcing its way through it, to the extreme disgust of the dentist. In this method, there is no gateage cut in anterior portion of case, so the pink cannot escape, nor can the other rubber enter the space already occupied.—Dr. H. S. Miller, in *Dental Brief*.

*Some Prosthodontic Criticisms.* After reading a paper and the discussion thereon at the May meeting of the Chicago-Odontographic Society, I find some points wherein I disagree with what was advocated at that time.

And first, as to the manner of scraping impressions and models, I have found from long experience that it is unnecessary and unsatisfactory. Take the impression, for instance; you have the jaw in reverse, while in the model the jaw is present. To scrape the impression to a uniform thickness, not too much nor too little, is a very unsatisfactory performance. On the other hand, with the jaw before you, a "relief" of ordinary baseplate wax can be easily applied of uniform thickness. This should extend nearly to the margin of the ridge, as it is very hard there; in fact, if there is a soft ridge the relief should be thicker there than farther back, as the flexible ridge yields to pressure. The relief should cover as much of the hard surface as possible, not extending on to the sides over the soft tissue. The margins should be flush with the model.

As to scraping the soft tissue on the model, that is uncalled for, because all that is needed is for the plate to come in contact, not to press upon it.

Scraping for the margins of the plate is unnecessary, especially upon the labial side. The plate should be worn as high as possible and over the cuspid eminence higher than elsewhere. Instead of scraping then, after fitting the plate, I turn the extreme margin out, perhaps the thirty-secondth of an inch, so it shall not impinge upon the soft tissues.

A correct impression, a correct model, a plate swaged upon a Babbit metal die, will fit that model, and if that model represents the jaw the plate will fit the jaw. It is along that line I have worked these many years with success. No new impressions, no new dies, no fitting with pliers and burnishers.

To sum up, the plate should rest upon the alveolar ridge, in close contact with the sides and back of the relief, which covers nearly all the hard tissue. Plates can be worn farther back than they are generally made.

The above principle applies to 98 per cent of cases. There are about 2 per cent of cases where the palatal surface is soft. In these cases no relief or vacuum cavities are needed, but simply fit the plate snugly to the palate.

As to the causes of excessive absorption of process, no mention was made of the effect produced by the non-conductivity of vulcanite. I claim that 80 per cent of upper jaws are sooner or later ruined from this cause alone. Practicing thirteen years before the use of vulcanite, exclusively in plate work, and using it ever since, I have had peculiar opportunity for observation of its effects. Within two years of its use, myself and Dr. Allport, with whom I was associated, noticed an unusual change in the process in the case of the vulcanite plates, and I have watched it ever since.

The statement is made that "plates should not be worn over five or six years." This is true of vulcanite, but not at all of metal plates. I expect a continuous gum dentine to last at least 20 years and have seen scores in wear 25 years and upwards. One of the first I made, 55 years ago, I saw in wear 40 years later. Two years ago I made a set for a lady 80 years of age, who was wearing a set I made her 45 years previous. Old as she was, she would have nothing different. A few years ago I visited an old patient in Boston, who was wearing a set on gold which she had 50 years. I was surprised to find what good condition the jaw was in. The temporary plate should never be worn over two years, as excessive absorption takes place.

Much has been written about the expansion of plaster, but I have never found that it interfered with success of the denture. Theoretically, what results from the expansion? In the case of the impression it can expand only into the opening, which results in contraction. In case of the model it cannot expand inwardly, because held by the walls of the impression tray. Theoretically, the model is smaller than the jaw.

As to varnish, sandarach is objectionable because it leaves a film; on the other hand, shellac, mixed thin, penetrates and leaves no film, therefore preferable both for impression and model. I find no occasion for using any powder on the model in molding. Soap the impression, washing out the excess.

Cores are not needed in one case in fifty, and should be made of plaster and asbestos. In ordinary undercuts shape the model flaring in all cases, so it will drop out of the mold, never to be lifted out. Set the model highest in front and slope the heel more; then the model slips out easily.

The Chase oiled sand is excellent, as it is always ready for use, packing with a potato masher, handle at the sides, using a ring 5 inches in diameter and 2½ inches deep. Babbitt metal has all of the requisites for a dental die.

The Hawes flask I abandoned fifty years ago as unnecessary. Prepare the model so it will drop out readily, never to be lifted out.

After all that is done otherwise, there are more misfits and failures from faulty articulation than from all other causes, and too much pains cannot be taken in the final adjustment in the mouth, using the thick articulating paper, English make.—Dr. Haskell, in *Review.*

*Tooth Powders and Washes* Any first-class pharmacy has all the facilities, in the way of mixers, sifters, etc., for making tooth powder just as accurately and scientifically as a proprietary concern. In fact, if your prescription was taken to the proper corner drug store, it would probably be compounded by a graduate in pharmacy, who would take as much professional pride in preparing it *secundum artem* as you or I would in preparing a cavity or setting a crown.

For a dentist to write his own prescriptions shows a knowledge of materia medica and therapeutics. It is broadening. It is professional.

For a dentist to prescribe a nostrum shows lack of knowledge. It is narrowing. It is not professional.

Furthermore, by doing the former, you are serving your patients better, both from a remedial and monetary standpoint.

Let me illustrate this last statement: For two or three years I have been prescribing, in the majority of cases the following tooth powder:

R

Calcii carbonatis praeceptitatae............ℨ vi.

Sodii perboratis..........................ℨ i.

Pulveris saponis..........................gr. xx.

Olei gualtheriae, U. S. P.................m. xii.

Mix.

My office assistant keeps copies of the original on hand. My druggists have the powder ready to dispense.

I wish to call your attention to another oxygen tooth powder that I consider superior to Calox, as it contains a larger amount of available oxygen than the Kirk formula. This is Magnesium Peroxide cum Creta of the British Pharmaceutical Codex (Magnesium Peroxide with Chalk), and contains:

| | Gm. or c. c. |
|---|---|
| Magnesium peroxide | 10.00 |
| Powdered castile soap | 2.50 |
| Menthol | 0.10 |
| Oil of rose | 0.25 |
| Oil of wintergreen | 0.50 |
| Precipitated chalk, to make | 100.00 |

Mix.

Also the following:

R
Strontii peroxidi.........................ℨ ss.
Calcii carbonatis precipitatae............ℨ vi.
Pulveris saponis..........................gr. xxv.
Mentholis.................................gr. i.
Olei menthae piperitae....................m ii.
Mix.

Strantium peroxide, like sodium perborate, gives off oxygen in acid neutral or alkaline solutions.

MILD ALKALINE WASH.

I consider the following mouth washes superior to any nostrum on the market:

R
Sodii borobenzoatis, N. F.................ℨ xii.
Resorcnolis...............................gr. lxxx.
Glycerini.................................ℨ iv.
Alcoholis.................................℥ ii.
Olei Menthæ piperitae.....................m iv.
Olei cinnamomi............................
Eucolypotis, aa...........................m viii.
Talci purificati, q. s....................
Aquae destilatae, ad......................o i.
Mix. Sig.—One part to 1 or 2 of water.

### MILD ACID WASH.

℞

| | |
|---|---|
| Thymolis | |
| Mentholis, aa | gr. ii. |
| Glyceriti boroglyceridi | ℥ i. |
| Olei pini sylvestris | m xv. |
| Alcoholis | ℥ i. |
| Talci purificati, q. s. | |
| Aquae destillatae, ad | ℥ viii. |

Mix. Sig.—One part to 2 or 3 of water.

### SODIUM PERBORATE WASH.

Sodium perborate makes an inexpensive and effectual mouth-wash. I prescribe it as follows: One-fourth teaspoonful dissolved in one-fourth glass of water, to be used during the day. Make fresh each morning. If patient objects to the taste, a drop of an essential oil may be added to each mix.

Sodium perborate, when dissolved in water, decomposes into $H_2O_2$, and Sodium borate. In other words, we get a freshly prepared alkaline solution of hydrogen dioxide.

### LIQUOR ANTISEPTICUS. U. S. P.

Containing 2 percent, boric acid, 1 percent. benzoic acid and thymol, eucalyptol, essential oils and 25 percent. alcohol.

### LIQUOR ANTISEPTICUS ALKALINUS. N. F.

Containing glycerine, potassium bicarbonate, sodium benzoate, sodium borate, thymol, eucalyptol, oil of gaultheria, and peppermint and coloring.

### GLYCERINUM THYMOL COMPOSITUM OF THE BRITISH PHARMACEUTICAL CODEX.

(*Compound Glycerine of Thymol.*)

| | Gm. or c. c. |
|---|---|
| Sodium bicarbonate | 1.00 |
| Sodium borate | 2.00 |
| Sodium benzoate | 0.75 |
| Sodium salicylate | 0.50 |
| Menthol | 0.03 |
| Thymol | 0.05 |
| Eucalyptol | 0.13 |
| Oil of pine | 0.05 |
| Oil of wintergreen | 0.03 |
| Alcohol | 2.50 |
| Glycerine | 10.50 |
| Solution of carmine | 0.50 |
| Distilled water, to make | 100.00 |

Wadsworth tells us that dilute alcohol with normal salt solution, sodium bicarbonate, and glycerine, makes a most effectual mouth wash.

I submit the following:

R
Sodii chloridi..........................gr.xxxv.
Sodii bicarbonatis......................gr.xx .
Glycerini...............................ʒii
Alcoholis...............................℥ivss.
Aquae menthae piperitae.................℥ii
Aquae cinnamomi, ad.....................℥viii.
Mix. Sig.—Use with equal parts of water.

—Dr. G. B. Squires, in *Journal of Allied Sciences.*

